# ICTV Virus Taxonomy Profile: Megabirnaviridae

**DOI:** 10.1099/jgv.0.001297

**Published:** 2019-08-16

**Authors:** Yukiyo Sato, Naoyuki Miyazaki, Satoko Kanematsu, Jiatao Xie, Said A. Ghabrial, Bradley I. Hillman, Nobuhiro Suzuki, ICTV Report Consortium

**Affiliations:** 1 Institute of Plant Science and Resources, Okayama University, Chuo 2-20-1, Kurashiki 710-0046, Japan; 2 Life Science Center for Survival Dynamics, Tsukuba Advanced Research Alliance, University of Tsukuba, Tsukuba, Ibaraki 305-8577, Japan; 3 National Agriculture and Food Research Organization (NARO) Headquarters, 3-1-1 Kannondai, Tsukuba 305-8517, Japan; 4 College of Plant Science and Technology, Huazhong Agricultural University, Wuhan, PR China; 5 Department of Plant Pathology, University of Kentucky, Lexington, KY 40546, USA; 6 Department of Plant Biology and Pathology, Rutgers University, New Brunswick, NJ 08901, USA

**Keywords:** ICTV Report, taxonomy, *Megabirnaviridae*

## Abstract

*Megabirnaviridae* is a family of non-enveloped spherical viruses with dsRNA genomes of two linear segments, each of 7.2–8.9 kbp, comprising 16.1 kbp in total. The genus *Megabirnavirus* includes the species *Rosellinia necatrix megabirnavirus 1*, the exemplar isolate of which infects the white root rot fungus (*Rosellinia necatrix*) to which it confers hypovirulence. Megabirnaviruses are characterized by their bisegmented genome with large 5′-untranslated regions (1.6 kb) upstream of both 5′-proximal coding strand ORFs, and large protrusions on the particle surface. This is a summary of the ICTV Report on the family *Megabirnaviridae*, which is available at ictv.global/report/megabirnaviridae.

This Profile is dedicated to the memory of our valued colleague Professor Said A. Ghabrial.

## Virion

**Fig. 1.**
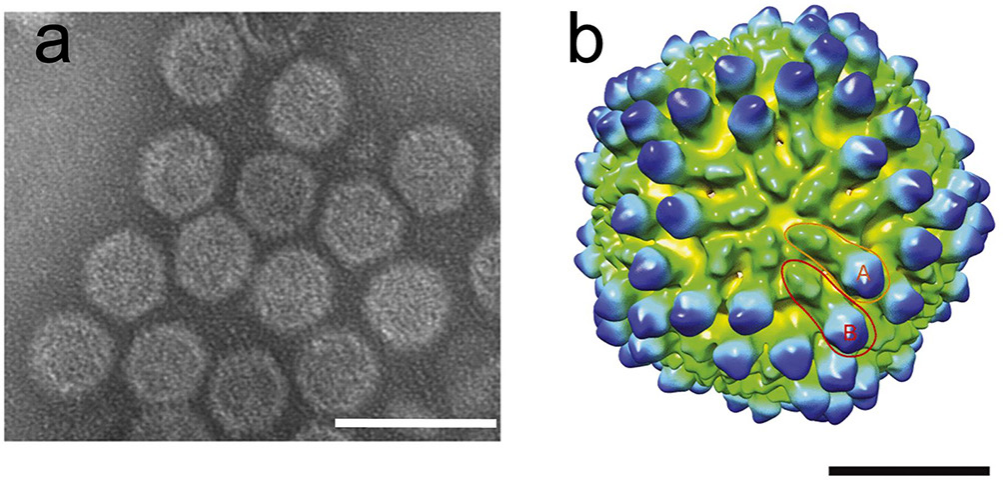
Megabirnavirus particle structure – Rosellinia necatrix megabirnavirus 1-W779. (**a**) Transmission electron micrograph of negatively-stained virions. Scale bar, 100 nm. Reproduced with permission from [1]. (**b**) Surface representation of virion reconstructed by three-dimensional cryo-EM at a resolution of 15.7 Å. Scale bar, 20 nm. Reproduced with permission from [2].

**Fig. 2.**
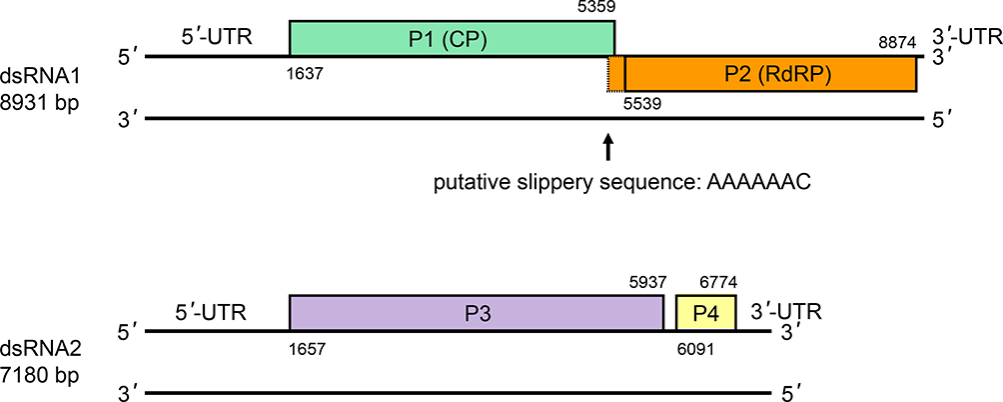
Genomic organization of Rosellinia necatrix megabirnavirus 1-W779. Black lines represent genomic dsRNA segments. Boxes indicate ORFs on the positive-sense strands with dotted lines indicating the start of the -1 frameshifted reading frame .

Megabirnaviruses form rigid spherical particles with a diameter of 52 nm (Table 1, Fig. 1a) [1,2]. Each capsid with a *T*=1 lattice is composed of 60 asymmetric homodimers of the capsid protein, P1 (Fig. 1b), which are presumed to produce 120 protrusions on the virus surface. The genomic segments, dsRNA1 and dsRNA2, appear to be packaged into separate particles. The major capsid protein is encoded by the 5′-proximal open reading frame (ORF) on dsRNA1, which also encodes an RNA-directed RNA polymerase (RdRP) in the 3′-proximal ORF (Fig. 2). Purified virions include capsid protein-RdRP fusion protein, which is translated from polycistronic mRNA transcribed from dsRNA1 [1,3]. Purified virions are infectious to protoplasts of natural and experimental fungal hosts [1,3, 4].

**Table 1.** Characteristics of members of the family *Megabirnaviridae*

Typical member:	Rosellinia necatrix megabirnavirus 1-W779 (RNA1: AB512282; RNA2: AB512283), species *Rosellinia necatrix megabirnavirus 1*, genus *Megabirnavirus*
**Virion**	Isometric, non–enveloped particles, 52 nm in diameter; dsRNA segments may be separately encapsidated
**Genome**	Two linear dsRNAs of 7.2–8.9 kbp, 16.1 kbp in total; large untranslated regions (over 1.6 kb) upstream of both 5′-proximal coding strand ORFs
**Replication**	Possibly within virus particles, as observed for other dsRNA viruses
**Translation**	Possible internal ribosomal entry site translation of the 5′-proximal ORFs on the mRNAs from dsRNA1and dsRNA2. The 3′-proximal ORF of dsRNA1 is translated via −1 ribosomal frameshifting
**Host range**	Fungi
**Taxonomy**	Realm *Riboviria*, one genus including a single species

## Genome

The genome of Rosellinia necatrix megabirnavirus 1-W779 consists of two segments, dsRNA1 and dsRNA2 (8.9 kbp and 7.2 kbp, respectively) (Fig. 2) [1]. Each segment has two ORFs; dsRNA1 encodes the capsid (P1) and RdRP (P2) proteins, while dsRNA2 encodes the hypothetical proteins, P3 and P4, with unknown functions. The 5′-proximal ORFs are preceded by large (over 1.6 kb) untranslated regions. There is a putative slippery sequence (5′-AAAAAAC-3′) immediately before the stop codon of the P1 ORF, followed by a sequence that is predicted to form a potential stem–loop structure. This suggests that the capsid protein-RdRP fusion protein is likely to be produced via −1 ribosomal frameshifting.

## Taxonomy

The exemplar isolate of *Rosellinia nexatrix megabirnavirus 1*, a member of the genus *Megabirnavirus*, is Rosellinia necatrix megabirnavirus 1-W779, which was isolated from a phytopathogenic ascomycetous fungus that causes white root rot in many perennial crops worldwide. The mycoviruses Sclerotinia sclerotiorum megabirnavirus 1 and Rosellinia necatrix megabirnavirus 2 are closely related to Rosellinia necatrix megabirnavirus 1 but are currently unclassified [5,6]. Other mycovirus sequences have been detected from diverse ascomycetous and basidiomycetous fungi [7,10], and have deduced RdRP amino acid sequences with 30–47 % identity to that of Rosellinia necatrix megabirnavirus 1.

## Resources

Full ICTV Report on the family *Megabirnaviridae*: ictv.global/report/megabirnaviridae.

## Abbreviations

RdRP: RNA-directed RNA polymerase
